# Risk and protective factors for child development: An observational South African birth cohort

**DOI:** 10.1371/journal.pmed.1002920

**Published:** 2019-09-27

**Authors:** Kirsten Ann Donald, Catherine J. Wedderburn, Whitney Barnett, Raymond T. Nhapi, Andrea M. Rehman, Jacob A. M. Stadler, Nadia Hoffman, Nastassja Koen, Heather J. Zar, Dan J. Stein

**Affiliations:** 1 Department of Paediatrics and Child Health, Red Cross War Memorial Children’s Hospital and University of Cape Town, Cape Town, South Africa; 2 Neuroscience Institute, University of Cape Town, Cape Town, South Africa; 3 Department of Clinical Research, London School of Hygiene & Tropical Medicine, London, United Kingdom; 4 Unit on Child and Adolescent Health, South African Medical Research Council (SAMRC), Cape Town, South Africa; 5 Department of Infectious Disease Epidemiology, London School of Hygiene & Tropical Medicine, London, United Kingdom; 6 Department of Psychiatry and Mental Health, University of Cape Town, South Africa; 7 Unit on Risk and Resilience in Mental Disorders, South African Medical Research Council (SAMRC), Cape Town, Cape Town, South Africa; University College London, UNITED KINGDOM

## Abstract

**Background:**

Approximately 250 million (43%) children under the age of 5 years in low- and middle-income countries (LMICs) are failing to meet their developmental potential. Risk factors are recognised to contribute to this loss of human potential. Expanding understanding of the risks that lead to poor outcomes and which protective factors contribute to resilience in children may be critical to improving disparities.

**Methods and findings:**

The Drakenstein Child Health Study is a population-based birth cohort in the Western Cape, South Africa. Pregnant women were enrolled between 20 and 28 weeks’ gestation from two community clinics from 2012 to 2015; sociodemographic and psychosocial data were collected antenatally. Mothers and children were followed through birth until 2 years of age. Developmental assessments were conducted by trained assessors blinded to background, using the Bayley-III Scales of Infant and Toddler Development (BSID-III), validated for use in South Africa, at 24 months of age. The study assessed all available children at 24 months; however, some children were not able to attend, because of loss to follow-up or unavailability of a caregiver or child at the correct age. Of 1,143 live births, 1,002 were in follow-up at 24 months, and a total of 734 children (73%) had developmental assessments, of which 354 (48.2%) were girls. This sample was characterised by low household employment (*n* = 183; 24.9%) and household income (*n* = 287; 39.1% earning <R1,000 per month), and high prevalence of maternal psychosocial risk factors including alcohol use in pregnancy (*n* = 95; 14.5%), smoking (*n* = 241; 34.7%), depression (*n* = 156; 23.7%), lifetime intimate partner violence (*n* = 310; 47.3%), and history of maternal childhood trauma (*n* = 228; 34.7%). A high proportion of children were categorised as delayed (defined by scoring < −1 standard deviation below the mean scaled score calculated using the BSID-III norms from a United States population) in different domains (369 [50.5%] cognition, 402 [55.6%] receptive language, 389 [55.4%] expressive language, 169 [23.2%] fine motor, and 267 [38.4%] gross motor). Four hundred five (55.3%) children had >1 domain affected, and 75 (10.2%) had delay in all domains. Bivariate and multivariable analyses revealed several factors that were associated with developmental outcomes. These included protective factors (maternal education, higher birth weight, and socioeconomic status) and risk factors (maternal anaemia in pregnancy, depression or lifetime intimate partner violence, and maternal HIV infection). Boys consistently performed worse than girls (in cognition [β = −0.74; 95% CI −1.46 to −0.03, *p* = 0.042], receptive language [β = −1.10; 95% CI −1.70 to −0.49, *p* < 0.001], expressive language [β = −1.65; 95% CI −2.46 to −0.84, *p* < 0.001], and fine motor [β = −0.70; 95% CI −1.20 to −0.20, *p* = 0.006] scales). There was evidence that child sex interacted with risk and protective factors including birth weight, maternal anaemia in pregnancy, and socioeconomic factors. Important limitations of the study include attrition of sample from birth to assessment age and missing data in some exposure areas from those assessed.

**Conclusions:**

This study provides reliable developmental data from a sub-Saharan African setting in a well-characterised sample of mother–child dyads. Our findings highlight not only the important protective effects of maternal education, birth weight, and socioeconomic status for developmental outcomes but also sex differences in developmental outcomes and key risk and protective factors for each group.

## Abstract 2

Kirsten Ann Donald and colleagues reveal the protective effects of birth weight, mother's education, and socioeconomic status in a child's development in South Africa.

## Author summary

**Why was this study done?:**

- Child development in early childhood lays a foundation for lifelong learning.
- Risk and protective factors for child development are known to include many issues faced by children growing up in low- and middle-income countries.
- Studies indicate a difference between boys and girls in terms of impact of factors influencing development, but these have not been evaluated in a sub-Saharan African context.

**What did the researchers do and find?:**

- We assessed child development at 2 years of 734 children in the Drakenstein Child Health Study, Western Cape, South Africa.
- We assessed potential risk and protective factors identified from prior literature to impact child development.
- We found a number of important risk factors that contributed to poor developmental outcomes in children in this cohort.

**What do these findings mean?:**

- Boys appear to be at higher risk of poor developmental performance in a high-risk environment.
- Key protective factors include mothers having at least some secondary school education, better home circumstances, and healthy birth weight, and key risk factors include maternal anaemia in pregnancy, poor maternal health (such as HIV), and maternal mental health problems.
- Child sex interacts with the associations between key protective and risk factors and developmental outcomes.
- Understanding the related and interacting roles of factors reported in this study may inform integrated intervention policy design and implementation for supporting development in high-risk environments.

## Introduction

Approximately 250 million (43%) of children under the age of 5 years in low- and middle-income countries (LMICs) are at risk of poor developmental outcomes [1]. Child development takes place as an ongoing biological and psychological process influenced by the environment, caregivers, community, and society. Key risk factors known to affect child development may be broadly grouped into those affecting (1) the wider community and environment in which the child and family live, often termed the social determinants of health [2] (poverty, lack of access to education, environmental stressors, poor water and sanitation); (2) the physical health of the caregiver [3] (maternal illness and nutrition) and the child (malnutrition, low birth weight, infections); (3) and maternal psychosocial health [4] (maternal depression, substance use, and intimate partner violence [IPV]). Conversely, protective factors are those that foster resilience and allow children to overcome adversity. These include (1) breastfeeding and good nutrition, (2) clean and safe living spaces, (3) and nurturing environments and healthy parents [3,5]. Improved understanding of key factors associated with neurodevelopmental delay and those promoting resilience is necessary to ensure children achieve their developmental potential. Children exposed to multiple risk factors have a greater likelihood of poor adult health and well-being [6].

There is increasing recognition that boys and girls may be sensitive to their environments in different ways. Previous work has further indicated an emerging theme of sex-dependent fetal programming in response to prenatal stress of various types, whereby girls may respond to challenge in more anxious and reactive ways and boys respond in less reactive but more aggressive ways [7]. More specifically, studies in high-income countries have reported a difference in developmental outcomes between girls and boys [8], and recently, a multicountry evaluation highlighted large disparities between sexes in the Asia-Pacific region, with girls performing better than boys on composite developmental score in four out of six countries [9]. This supports the hypothesis that boys and girls may be differentially affected by risk and protective factors impacting their future development [10]. However, very little of this work has been done in LMICs (and has not been explored in sub-Saharan Africa), where there is amplified potential of multiple physical, psychosocial, and environmental factors to interact in complex ways and to which boys and girls may be vulnerable in different ways.

Data are lacking from LMICs that comprehensively investigate the development of children including cognitive, language, and motor outcomes. Although recently there have been some trials examining risk factors for development [11], global estimations tend to use proxy measures, such as poverty and stunting, as measures of child development [1] because of insufficient data directly measuring these outcomes and risk factors in LMIC contexts. This gap in the literature highlights the need to report broad, multifactorial (social, clinical, psychosocial) data measuring risk and protective factors for early child development and appropriate, directly measured developmental outcomes in children living in these settings. Expanding our understanding of which risk factors lead to poor outcomes and which protective factors build resilience is critical to improving disparities, particularly in low-resource settings [12,13]. The aim of this study was to investigate the range of risk and protective factors that affect early childhood developmental outcomes and to determine sex differences in the impact of such factors in a South African birth cohort focussed on early child health and development.

## Methods

### Site

The Drakenstein Child Health Study (DCHS) is a multidisciplinary population-based birth cohort study investigating the early-life determinants of child health and development. The study is located in Paarl, a periurban area, 60 km outside of Cape Town in the Western Cape of South Africa [14]. It is a stable, low-socioeconomic community comprising approximately 200,000 people, characterised by a high prevalence of a range of health risk factors such as depression, childhood trauma, IPV, and poverty [15]. The DCHS is representative of many periurban regions in South Africa, as well as in other LMICs. The majority of the population accesses healthcare in the public sector. Pregnant women were recruited from two public sector primary healthcare clinics, one serving a predominantly mixed ancestry population (TC Newman) and the other serving a predominantly black African population (Mbekweni).

### Population

Pregnant women were enrolled into the DCHS between 20 and 28 weeks’ gestation while attending routine antenatal care and are being prospectively followed through childbirth and early childhood until children are 10 years of age. Recruitment was unfiltered, and eligibility criteria included (1) attendance at one of the two study clinics, (2) being at least 18 years of age, and (3) intending to remain in the study area for at least 1 year. Between March 2012 and March 2015, 1,225 pregnant women were enrolled into the DCHS antenatally; 88 (7.2%) mothers were lost to follow-up antenatally, had a miscarriage, or had a stillbirth. In total, there were 1,143 live infants.

### Measures

All measures were performed as part of the main study, and child development outcomes form a primary aim of the original DCHS [14,15]. Expanded descriptive detail and rationale may be found in the associated Methods paper [16].

### Sociodemographic and environmental variables

Sociodemographic variables were measured using validated questionnaires administered by trained study staff at an antenatal visit at 28 to 32 weeks’ gestation. Sociodemographic variables including household factors (running water, flushing toilet, electricity in home, and household income) and maternal demographics (age at enrolment, any secondary versus only primary education, married or cohabiting with partner, employed, and whether this was the first pregnancy) were collected using an interviewer-administered questionnaire adapted from items used in the South African Stress and Health (SASH) Study [17,18].

### Child and maternal physical health

Gestational age at delivery was calculated in weeks, based on ultrasound results when these were available and otherwise based on fundal height measurements and maternal report of last menstrual period. Prematurity was defined as <37-week gestational age. Birth weight was abstracted by trained study staff from hospital records at birth and was taken as a continuous measure. Duration of exclusive breastfeeding was derived based on maternal report of feeding practices at birth; 6, 10, and 14 weeks; and 6 and 9 months of child age. Exclusive breastfeeding was defined as occurring until the first maternal report of introduction of solid foods or formula. We included exclusive breastfeeding for 6 months, as per current recommendations by WHO [19].

Maternal haemoglobin was tested for in pregnancy. Haemoglobin levels < 10 g/dL in pregnancy were classified as moderate to severe iron deficiency anaemia as per WHO guidelines [20]. Maternal HIV status was established during routine HIV testing of women in pregnancy as per the Western Cape of South Africa guidelines for prevention of mother-to-child transmission of HIV. Stunting was investigated separately because of the known association with delayed development, but it was not included as a risk factor in the final model, because of the association with the other risk and protective factors [21]. Stunting was defined here as <−2 standard deviations below WHO z-score height for age at 2 years of age.

### Substance use

Alcohol use during pregnancy was assessed using a composite, dichotomous measure (exposure versus no exposure) using the Alcohol, Smoking and Substance Involvement Screening Test (ASSIST) and retrospectively collected data on hazardous alcohol use during pregnancy [22,23]. The ASSIST has shown good reliability and validity in international, multisite studies. Total scores were obtained for alcohol by summing individual items related to maternal alcohol use during pregnancy; a score of >10 indicates moderate to high levels of risk for alcohol problems (reflecting weekly or daily/almost daily alcohol use and negative consequences related to the quantity of alcohol consumed). We categorised children as alcohol-exposed versus not alcohol-exposed. We used the following inclusion criteria to define alcohol exposure in order to optimise numbers: (1) scoring greater than moderate on the ASSIST performed antenatally as per reference Myers et al. [22] and Donald et al. [23] AND/OR (2) 2 or more drinks a week on the neonatal alcohol questionnaire (to further quantify alcohol use on top of the ASSIST) AND/OR (3) 2 or more drinks a week on a retrospective alcohol questionnaire regarding alcohol use during pregnancy (as mothers may be more likely to respond to this than during/immediately after pregnancy). Tobacco exposure during pregnancy was objectively measured by maternal urine cotinine collected antenatally or at birth. Cotinine, a metabolite of nicotine measurable in the urine, was measured using the IMMULITE 1000 Nicotine Metabolite Kit (Siemens Medical Solutions Diagnostics, Glyn Rhonwy, Llanberis, United Kingdom). Mothers were categorised as active smokers during pregnancy if either the antenatal or birth cotinine levels were ≥500 ng/ml [18].

### Psychosocial measures

Mothers completed a battery of psychosocial measures, administered by trained study staff, at an antenatal visit between 28 and 32 weeks’ gestation. The IPV questionnaire used in this study was adapted from WHO’s multicountry study [24] and the Women’s Health Study in Zimbabwe [25]. Mothers were asked about exposure to partner behaviour and frequency of occurrence for emotional, physical, or sexual abuse behaviours; when behaviours were experienced ‘many times’, mothers were categorised as exposed to lifetime IPV. The Edinburgh Postnatal Depression Scale (EPDS) [26] was used to measure depression; this scale has been validated for use with pregnant women and in a South African population [27–29]. The EPDS consists of 10 items referring to the past 7 days; a total score was obtained by summing responses for all items, and a cutoff score of ≥13 was used to dichotomise participants into below and above threshold for depression. The Childhood Trauma Questionnaire [30] Short-Form was used to assess abuse and neglect experienced as a child. Each item was responded to on a 5-point scale; a cutoff score of >36 was used to dichotomise mothers into above or below threshold for childhood abuse. The Self Reporting Questionnaire-20 (SRQ-20) is a WHO-endorsed measure of psychological distress [31]. The SRQ-20 consists of 20 items, which assess nonpsychotic symptoms, including symptoms of depressive and anxiety disorders; items are summed, and a cutoff score of >8 was used to dichotomise mothers into below or above threshold for psychological distress [32].

### Child development: Bayley Scales of Infant and Toddler Development

Child development was assessed on all available children across the full cohort at 24 months of age using the Bayley Scales of Infant and Toddler Development (Third Edition) (BSID-III) [33]. The BSID-III is a gold-standard observational measure of development for children from 0 to 42 months. It has been validated for a South African population [34,35] and found to be culturally appropriate without modifications. However, the tool may slightly underestimate delay in this population [35]. The tool measures development by direct observation across five subscales: cognition, receptive and expressive language, and fine and gross motor [36]. These scales were measured by direct observation by a trained physiotherapist and occupational therapist blinded to the child and family risk factors, overseen by a paediatric neurologist with specialist developmental expertise [16]. Quality control and monitoring processes were implemented to ensure accuracy. All data were entered into the BSID-III scoring programme, and the data were exported to Excel. In this analysis, both the raw scores and scaled scores were used. The scaled scores are calculated using a normal US population, scaled to a mean of 10 and standard deviation of 3. We assessed poor developmental outcomes [33] by categorising the scores into ‘delay’ or ‘no delay’, defined by scoring <−1 standard deviation below the mean scaled score (using a cutoff of 7).

### Ethical considerations

Ethical approval was obtained from the Faculty of Health Sciences Research Ethics Committee, University of Cape Town (401/2009), and by the Western Cape Provincial Research committee (2011RP45). Mothers gave written informed consent at enrolment and were reconsented annually for study involvement. Consent was done in the mother’s preferred language: English, Afrikaans, or isiXhosa. When significant developmental delays were identified by study staff, mothers and children were referred to local healthcare services for further assessment and management.

### Statistical strategy

The analyses followed a defined approach that was decided upon prior to running the models, though no prespecified analysis plan exists. Thus, data-driven approaches to analysis were not used at any stage. Early childhood development outcomes’ scores were measured by five developmental domains using the BSID-III: cognitive development, language (receptive and expressive) development, and motor (fine and gross) development. We used a cutoff scaled score of less than 7 (<−1 SD below the mean scaled score) to define developmental delay.

**Fig 1:**
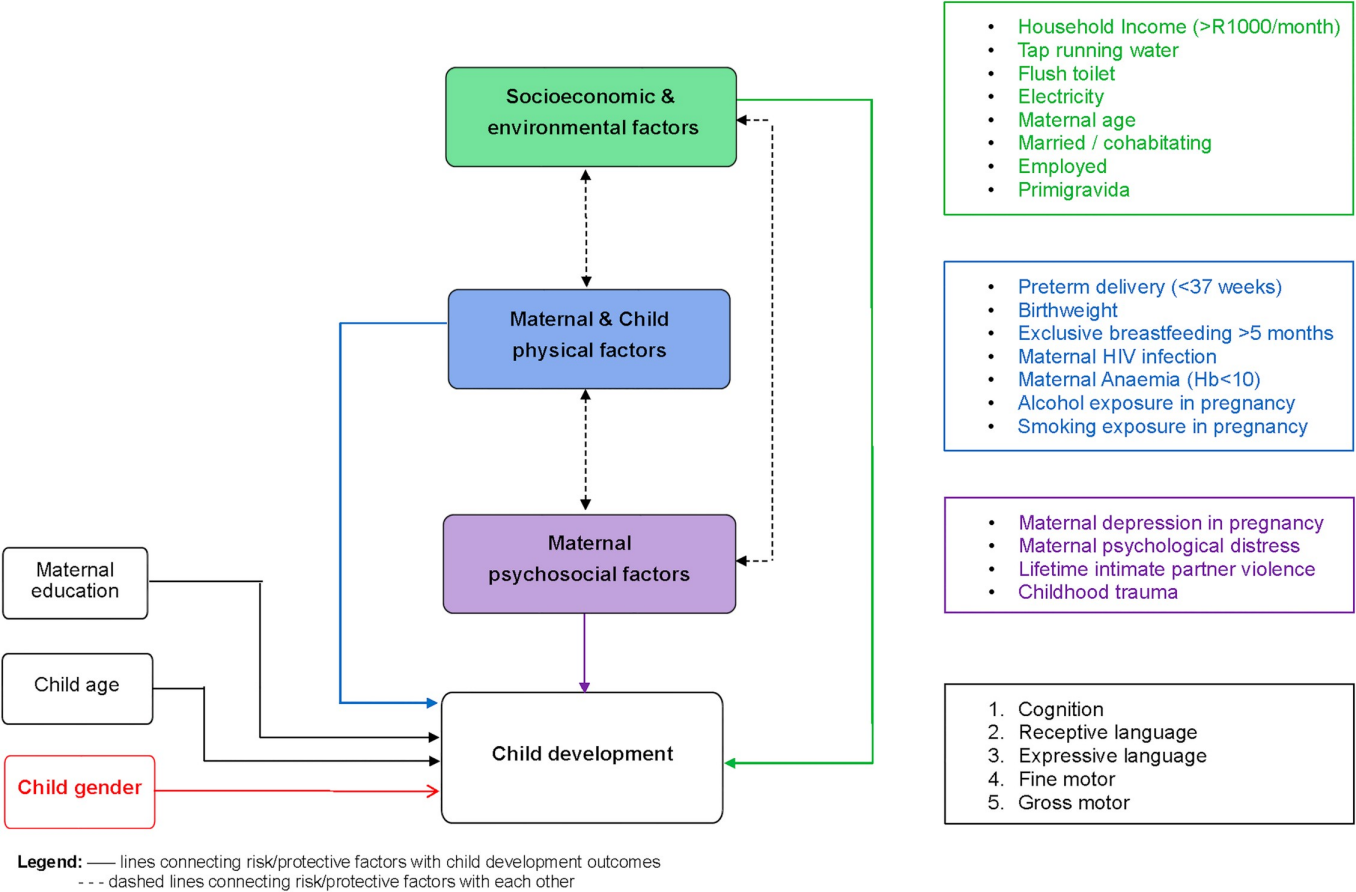
Methodological conceptual framework: Hierarchical model. This was adapted from the model by Chopra and colleagues [36].

BSID-III raw scores were analysed using linear regression, and developmental-delay data were analysed using logistic regression separately for each domain. Odds ratios are presented for logistic regression models, and regression coefficients are presented for linear regression models with 95% confidence intervals (CIs) for both. We used a missing-at-random approach and generated a complete-case dataset, which omitted records that were missing data in any of the covariates that we considered plus any domain (apart from gross motor, which was not analysed). Multivariable regression models were built using the complete-case dataset in a hierarchical approach. Initial models adjusted for maternal education, child age, and sex forced into the model as known and well-proven factors that impact child developmental outcomes. Subsequently, other covariates were added in blocks following the hierarchical order from sociodemographic and environmental variables to maternal and child physical variables to maternal psychosocial variables based on prior literature [37] as illustrated in Fig 1. Covariates added from each block were selected using a best-subsets variable-selection approach that aimed to minimise Akaike information criterion (AIC; using the ‘gvselect’ command in Stata 14) [38,39]. That is, the model for one outcome considered forced variables first and compared the fit of the model to the fit of the models with all possible combinations of variables from the sociodemographic block. Only the subset of these variables that reduced the AIC were retained for consideration with the next block (this includes an empty set, which would arise if no variables reduced the AIC from the previous model). Collinearity was assessed using variance inflation factors after each block, and then these steps were repeated for the next two blocks of variables. After creating a final model assessing the associations between risk and protective factors and each developmental outcome, we assessed whether including interaction terms between child sex and any variable retained from the best-subsets regression reduced the AIC further, to explore whether child sex altered the association between any of these variables and developmental outcomes. We report both the final model and the model including interaction terms. Finally, we used the same process to assess the outcome of global developmental delay in all domains consisting of delay in cognition, language (combining delay in receptive or expressive language), fine motor, and gross motor.

All analyses were performed with Stata version 14 [40].

## Results

Of 1,143 live study births, 409 (35.8%) children were lost to follow-up before reaching 2 years of age or did not complete a BSID-III assessment, because of nonattendance or moving out of the study area. Therefore, a total of 734 children completed BSID-III assessments at 2 years of age between August 2014 and September 2017. There were few differences between those who did and did not complete a BSID-III at 2 years (S1 Table), including higher maternal age (*p* < 0.01), less prematurity (*p* < 0.01), and more active smoking in pregnancy (*p* = 0.01) in those with a BSID-III.

Baseline clinical, demographic, and psychosocial characteristics, stratified by child sex, are presented in Table 1. Of the children with a BSID-III, 48.2% were girls, and there were no differences in sociodemographic variables between sexes (*p* < 0.05), apart from girls having a higher household income (65.5% had >R1,000 [approximately USD 100] per month versus 56.6% boys, *p* = 0.01) (Table 1). This sample was characterised by low household employment (24.9%) and low household income (39.1% earned less than R1,000 per month). The majority of households had running water (69.2%) and electricity (95.0%). A minority of mothers were married or cohabiting (40.1%), one-third of babies were born to primigravid mothers (33.0%), and 14.2% of babies were born prematurely (<37 weeks’ gestation). Of the 169 mothers (23.0%) who were confirmed HIV-infected antenatally, one child tested HIV-positive. Breastfeeding rates were low across the cohort, with only 17.1% children exclusively breastfed for 6 months. There was a high proportion of antenatal maternal substance use and other psychosocial risk factors, including alcohol exposure (14.5%), smoking (34.7%), depression (23.7%), lifetime IPV (47.3%), and history of maternal childhood trauma (34.7%).

**Table 1:** Demographics and baseline characteristics for those children completing at least one domain of the BSID-III.

* *Variables, *n* (%)	Female	Male	Total
*N*	354	380	734
Child sex	354 (48.23)	380 (51.77)	734 (100)
Maternal education: Secondary and above	326 (92.09)	348 (91.58)	674 (91.83)
Child age (mean, SD)	24.07 (0.49)	24.07 (0.55)	24.07 (0.52)
**Socioeconomic variables**			
Household income > R1,000 (approximately USD 100) per month	232 (65.54)	215 (56.58)	447 (60.90)
Tap running water	254 (71.95)	252 (66.67)	506 (69.22)
Flush toilet	236 (66.86)	230 (60.69)	466 (63.66)
Electricity	335 (94.90)	360 (94.99)	695 (94.95)
Maternal age at enrolment (mean, SD)	27.14 (5.78)	26.82 (5.87)	26.97 (5.83)
Married or cohabitating	152 (43.06)	142 (37.37)	294 (40.11)
Employed	89 (25.14)	94 (24.74)	183 (24.93)
Primigravida	111 (31.36)	131 (34.47)	242 (32.97)
**Physical variables**			
Preterm	49 (13.88)	55 (14.51)	104 (14.21)
Birth weight (kg) (mean, SD)	3.00 (0.589)	3.09 (0.60)	3.05 (0.59)
Exclusive breastfeeding for 6 months	61 (17.28)	64 (16.84)	125 (17.05)
Maternal HIV infection	74 (20.90)	95 (25.00)	169 (23.02)
Maternal anaemia in pregnancy	62 (18.56)	54 (15.61)	116 (17.06)
Maternal alcohol use in pregnancy	42 (13.33)	53 (15.54)	95 (14.48)
Maternal active smoking in pregnancy	111 (33.33)	130 (36.01)	241 (34.73)
**Psychosocial variables**			
Antenatal depression	78 (24.76)	78 (22.74)	156 (23.71)
Antenatal psychological distress	73 (23.17)	65 (18.95)	138 (20.97)
Lifetime intimate partner violence	143 (45.54)	167 (48.83)	310 (47.26)
Maternal childhood trauma	109 (34.60)	119 (34.69)	228 (34.65)

Missing data: Tap water (*n* = 3); flushing toilet, electricity (*n* = 2); married/cohabitating status (*n* = 1); preterm (*n* = 2); birth weight (*n* = 4); breastfeeding (*n* = 1); maternal anaemia in pregnancy (*n* = 54); maternal alcohol in pregnancy (*n* = 78); maternal smoking in pregnancy (*n* = 40); antenatal depression, psychological distress, and maternal childhood trauma (*n* = 76); lifetime intimate partner violence (*n* = 78).

Abbreviation: BSID-III, Bayley-III Scales of Infant and Toddler Development (Third Edition)

### Child development

A total of 369/731 children (50.5%) were categorised as having cognitive delay, 402/723 (55.6%) with receptive language delay, 389/702 (55.4%) with expressive language delay, 169/730 (23.2%) with fine motor delay, and 267/696 (38.4%) with gross motor delay (Table 2). Overall, 405 children from the 734 (55.2%) were classified as having delay in two or more domains; the combination of cognitive and language delay was the most common, with 304 children (41.4%). There were 75 children (10.2%) who were delayed in all four domains.

**Table 2:** Child developmental outcomes at 24 months of age for all children who completed at least one domain of the BSID-III (*n* = 734).

Developmental subscale	Total	Male	Female	*P*
**Cognition**				
Mean (SD)	55.48 (4.82)	55.12 (4.90)	55.87 (4.71)	0.02*
Poor cognitive outcome, ***n*** (%)	369 (50.48)	205 (54.09)	164 (46.59)	0.04*
**Receptive language**				
Mean (SD)	20.80 (3.72)	20.22 (3.60)	21.41 (3.76)	<0.01**
Poor receptive outcome, ***n*** (%)	402 (55.60)	243 (64.97)	159 (45.56)	<0.01**
**Expressive language**				
Mean score (SD)	24.08 (5.09)	23.24 (4.85)	24.99 (5.18)	<0.01**
Poor expressive outcome, ***n*** (%)	389 (55.41)	231(63.29)	158 (46.88)	<0.01**
**Fine motor**				
Mean (SD)	37.47 (3.16)	37.15 (3.25)	37.82 (3.03)	<0.01**
Poor fine motor outcome, ***n*** (%)	169 (23.15)	104 (27.37)	65 (18.57)	<0.01**
**Gross motor**				
Mean (SD)	53.25 (3.59)	53.30 (3.31)	53.19 (3.87)	0.69
Poor gross motor outcome, ***n*** (%)	267 (38.36)	136 (37.99)	131 (38.76)	0.84

Mean total raw score presented; poor outcomes are defined as <−1 standard deviation below the mean scaled score. Mann-Whitney *U* tests were used for a comparison of means and the chi-squared tests for poor cognitive outcome.

Missing data: cognition (*n* = 3); receptive language (*n* = 11); expressive language (*n* = 32); fine motor (*n* = 4); gross motor (*n* = 38).

*p < 0.05.

**p < 0.01.

Abbreviation: BSID-III, Bayley-III Scales of Infant and Toddler Development (Third Edition)

On raw scores, boys exhibited lower total scores than girls for all domains except gross motor on bivariate analyses (Table 2 and S2 Table), as well as in adjusted analyses in cognition (β = −0.74; 95% CI −1.46 to −0.03, *p* = 0.042), receptive language (β = −1.10; 95% CI −1.70 to −0.49, *p* < 0.001), expressive language (β = −1.65; 95% CI −2.46 to −0.84, *p* < 0.001), and fine motor (β = −0.70; 95% CI −1.20 to −0.20, *p* = 0.006) raw scores (Table 3). Likewise, boys were at increased risk of delay in all domains except gross motor (Table 2 and S3 Table) (*p* < 0.05) in bivariate analyses, and this association held when adjusting for confounders in the multivariable models (S4 Table): cognitive delay (adjusted odds ratio [aOR] 1.35; 95% CI 0.95–1.91, *p* = 0.098), receptive language delay (aOR 2.40; 95% CI 1.68–3.42, *p* < 0.001), expressive language delay (aOR 2.12; 95% CI 1.49–3.03, *p* < 0.001), and fine motor delay (aOR 1.92; 95% CI 1.25–2.95, *p* = 0.003).

**Table 3:** Multivariable linear regression results demonstrating the association of risk and protective variables with developmental domain outcome for total raw scores and exploring interaction with child sex.

	Cognition		Receptive language		Expressive language		Fine motor
	Final model	Interaction with child sex model	Final model	Interaction with child sex model	Final model	Interaction with child sex model	Final model
**A priori variables**							
Education: ≥Secondary	1.70 (0.45–2.95)	1.65 (0.40–2.89)	0.75 (−0.32 to 1.82)	0.67 (−0.39 to 1.73)	1.23 (−0.21 to 2.67)	1.50 (0.06–2.94)	0.65 (−0.23 to 1.52)
Child age	0.69 (0.00–1.37)	0.71 (0.03–1.39)	0.51 (−0.07 to 1.09)	0.53 (−0.05 to 1.11)	0.97 (0.20–1.74)	0.91 (0.15–1.67)	0.38 (−0.10 to 0.86)
Child sex: Boys	−0.74 (−1.46 to −0.03)	−5.91 (−9.92 to −1.90)	−1.10 (−1.70 to −0.49)	−5.30 (−8.72 to −1.88)	−1.65 (−2.46 to −0.84)	−2.77 (−4.36 to −1.17)	−0.70 (−1.20 to −0.20)
**Socioeconomic**							
Household income: >R1,000 per month					0.78 (−0.05 to 1.61)	0.80 (−0.02 to 1.62)	
Tap running water					−1.84 (−3.13 to −0.56)	−3.10 (−4.70 to −1.49)* 2.15 (0.38–3.91)**	
Flush toilet					1.62 (0.39–2.86)	1.67 (0.45–2.90)	
Electricity			1.15 (−0.38 to 2.67)	1.10 (−0.42 to 2.61)			
Maternal age							
Married or cohabitating							
Employed			0.29 (−0.41 to 0.99)	0.43 (−0.27 to 1.14)			
Primigravida					0.93 (0.06–1.80)	2.07 (0.82–3.31)* −2.18 (−3.88 to −0.48)**	
**Physical**							
Birth weight	1.61 (0.96–2.26)	0.68 (−0.29 to 1.64)* 1.69 (0.40–2.98)**	1.02 (0.47–1.57)	0.28 (−0.55 to 1.11)* 1.30 (0.19–2.40)**	0.79 (0.05–1.54)	0.92 (0.18–1.66)	0.70 (0.24–1.15)
Preterm							
Exclusive breastfeeding for 6 months							
Maternal HIV infection			−0.61 (−1.35 to 0.14)	−0.63 (−1.37 to 0.11)	−0.92 (−1.92 to 0.07)	−0.92 (−1.91 to 0.06)	
Maternal anaemia in pregnancy	−1.38 (−2.33 to −0.44)	−1.38 (−2.32 to −0.44)	−1.12 (−1.93 to −0.31)	−1.78 (−2.89 to −0.67)* 1.40 (−0.19 to 2.98)**	−1.11 (−2.18 to −0.04)	−1.99 (−3.46 to −0.52)* 1.97 (−0.11 to 4.06)**	
Maternal alcohol use in pregnancy							
Maternal active smoking in pregnancy					−0.71 (−1.58 to 0.16)	−0.65 (−1.52 to 0.21)	
**Psychosocial**							
Antenatal depression	−1.03 (−1.94 to −0.12)	−1.03 (−1.93 to −0.13)					
Antenatal psychological distress	0.93 (−0.04 to 1.90)	0.97 (0.00–1.93)					
Lifetime intimate partner violence							
Maternal childhood trauma							

The ‘final model’ describes the multivariable model assessing associations of risk and protective factors and each developmental outcome.

The ‘interaction with child sex model’ explored the interaction with child sex for each variable included in the final model and shows the model inclusive of those interactions that reduced the AIC. Interactions with sex did not reduce the AIC in the fine motor model and are therefore not included.

The complete-case dataset was used here (*n* = 539).

Green signifies a positive association, with *p* < 0.05; red signifies a negative association, with *p* < 0.05.

Coefficients and 95% confidence intervals presented for variables in each model.

*Beta coefficient from the interaction model of the variable main effect.

**Beta coefficient from the interaction model of the variable with male sex.

Abbreviation: AIC, Akaike information criterion.

At 2 years of age, 19.3% of children were classified as stunted (22.4% boys and 15.8% girls), and stunting was found to be associated with poor cognitive development (β = −1.13; 95% CI −2.10 to −0.15) and with cognitive developmental delay (OR = 1.77, 95% CI 1.18–2.65). As expected, across the group, children who were older tended to perform better in the developmental assessment, particularly in the cognitive and language subscales. However, the age window was very small, so the variance may not be meaningful in this analysis. In the gross motor domain, we investigated sex, education, and child age and we did not see any of the expected bivariate associations and therefore we did not run multiple regression analyses on this outcome variable.

### Risk and protective factors for child development

**Fig 2:**
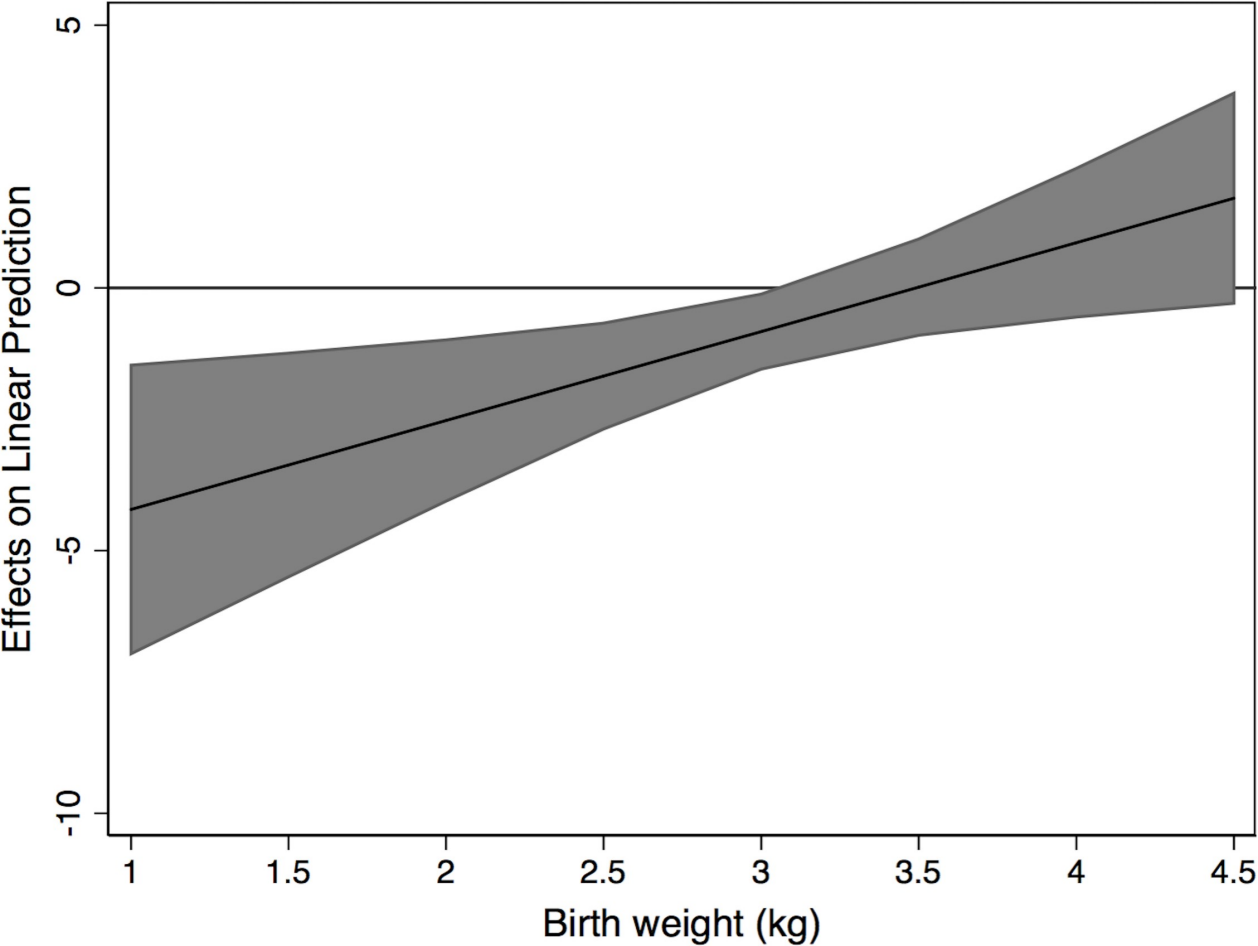
Marginal effects of birth weight and sex on cognition.

Bivariate analyses (S2 and S3 Tables) and the final model multiple regression results are shown in Table 3, S4 Table, Fig 2, and S1 Fig. We first describe the final model examining associations between risk and protective factors with child developmental outcomes and then explore the interactions with child sex.

### Sociodemographic and environmental factors

On bivariate analysis, protective factors associated with better development and reduced risk of delay comprised having higher maternal education, older child age, a primigravid mother, and better-resourced households (higher household income, flushing toilet, running water). Higher maternal education (any secondary versus only primary) was associated with increased total raw scores for all domains on bivariate analyses and in the final model for cognition (β = 1.70; 95% CI 0.45–2.95, *p* = 0.008) as well as lower odds of cognitive delay (aOR 0.52; 95% CI 0.28–0.99, *p* = 0.045) and receptive language delay (aOR 0.51; 95% CI 0.26–0.99, *p* = 0.045). Children of primigravid mothers had higher expressive language scores (β = 0.93; 95% CI 0.06–1.80, *p* = 0.036) and lower odds of expressive language delay (aOR 0.57; 95% CI 0.36–0.90, *p* = 0.016) in multiple regression models. In the interaction models, sex impacted the association between primigravida and expressive language outcomes. We calculated estimates of the interaction effects of primigravida in both boys and girls separately. For girls, the beta coefficient was 2.07 (95% CI 0.82–3.31, *p* = −0.01), and for boys, it was −2.88 (95% CI −4.64 to −1.12, *p* = 0.002). This suggests that primigravida status had a positive effect on expressive language for girls but a negative effect for boys in this cohort.

Flush toilets in household were associated with increased total raw scores for expressive language (β = 1.62; 95% CI 0.39–2.86, *p* = 0.010) in the multivariable regression. Tap water showed an interaction with child sex, and we calculated estimates of the effects of tap water in both boys and girls separately in the interaction model. For girls, the beta coefficient was −3.10 (95% CI −4.70 to −1.49, *p* < 0.001), and for boys, it was −3.72 (95% CI −5.41 to −2.02, *p* < 0.001). The effect direction is unexpected and is perhaps caused by the additional inclusion of flushing toilet in the model, although these together reduced the AIC.

### Mother and child physical factors

On bivariate analysis, key maternal and child physical health risk factors for lower developmental scores or higher odds of delay included maternal anaemia, prematurity, maternal HIV, alcohol or tobacco use during pregnancy, and maternal depression and lifetime IPV; protective factors included higher birth weight. Increasing birth weight was associated with significantly greater scores and lower odds for delay for all domains in the total cohort in bivariate and multivariable models (Table 3 and S4 Table). There was also evidence of an interaction with child sex in cognition and language outcomes. We calculated marginal effects for the interaction of birth weight and cognition, and at a birth weight of 3 kg (mean of cohort is 3.05 kg), the marginal effect was −0.83 (95% CI −1.55 to −0.12, *p* = 0.022). See Fig 2 for all marginal effects. At lower birth weights, boys did less well than girls (negative coefficients indicate a lower score); and at higher birth weights, boys did better. Likewise, we calculated marginal effects for the interaction of birth weight and receptive language, and at a birth weight of 3 kg, the marginal effect was −1.17 (95% CI −1.77 to −0.56, *p* < 0.001). See S1 Fig for all marginal effects. As with cognition, at lower birth weights boys did less well than girls.

In the multivariable final model, maternal anaemia in pregnancy was associated with lower cognitive scores (β = −1.38; 95% CI −2.33 to −0.44, *p* = 0.004), receptive language scores (β = −1.12; 95% CI −1.93 to −0.31, *p* = 0.007), and expressive language scores (β = −1.11; 95% CI −2.18 to −0.04, *p* = 0.043). There was some evidence for interaction of child sex in this association. We calculated estimates of the effects of anaemia in both boys and girls separately for receptive and expressive language. For girls (receptive language), the beta coefficient was −1.78 (95% CI −2.89 to −0.67, *p* = 0.002), and for boys, it was −5.68 (95% CI −9.25 to −2.11, *p* = 0.002). For girls (expressive language), the beta coefficient was −1.99 (95% CI −3.46 to −0.52, *p* = 0.008), and for boys, it was −2.79 (95% CI −4.79 to −0.78, *p* = 0.007). This suggests that anaemia in pregnancy had a greater negative effect on receptive and expressive language for boys than for girls, although it still had an effect in the girls. Maternal anaemia was also robustly associated with increased odds of cognitive delay (aOR1.66; 95% CI 1.04–2.66, *p* = 0.033), receptive language delay (aOR1.77; 95% CI 1.09–2.88, *p* = 0.020), and expressive language delay (aOR1.83; 95% CI 1.12–2.98, *p* = 0.015) overall. Maternal anaemia demonstrated a stronger interaction with these outcomes in boys as above.

### Maternal psychosocial factors

On bivariate analysis, maternal psychosocial risk factors associated with poorer developmental outcomes comprised maternal antenatal depression or lifetime maternal exposure to IPV. In multivariable regression, antenatal depression was associated with poorer cognitive scores (β = −1.03; 95% CI −1.04 to −0.12, *p* = 0.027). For maternal IPV, though the model without interactions was not significant in the adjusted analysis, there was an association with higher odds of expressive language delay, which came out in the interaction model (main effect aOR 1.88; 95% CI 1.13–3.13, *p* = 0.015).

### Developmental delay in all four domains

In bivariate analyses, maternal education and higher birth weight were protective against developmental delay in four domains, and preterm birth was associated with poorer outcomes.

On adjusted analyses, maternal education (aOR = 0.40; 95% CI 0.17–0.94, *p* = 0.035) was found to be protective against developmental delay in all four domains across the total cohort (S5 and S6 Tables)

## Discussion

This study provides directly measured developmental data from a low- and middle-income context in a well-characterised sample of mother–child dyads and emphasises the high prevalence of developmental delay. Our findings highlight the important protective effects of maternal education, birth weight, and socioeconomic status for developmental outcomes. Key risk and protective factors impacted developmental outcomes for boys and girls in different ways, and boys consistently performed worse than girls.

The substantial prevalence of developmental delay in around half the cohort reported in this study is higher than in previous reports. UNICEF’s caregiver-reported Early Childhood Development Index found that 37% of 3- and 4-year-olds in 35 LMICs do not attain basic cognitive and socio-emotional skills [41]. In this study, boys performed uniformly poorly compared to girls with lower raw scores across cognitive, language, and fine motor domains and correspondingly had increased risk of developmental delay in any one of these domains. This pattern of findings is consistent with other studies exploring developmental performance in very young children and has recently been described in a large multicountry study of older children across South East Asia [9].

In our cohort, key factors associated with positive development at 24 months included better socioeconomic status, higher birth weight, and higher maternal educational attainment. Maternal educational attainment was the strongest protective factor for reducing the odds of four-domain developmental delay across this group of vulnerable children. The importance of social determinants on child development is well described. In a recently published monograph reflecting on the Young Lives study spanning three different LMIC countries on three continents (Vietnam, Peru, and Ethiopia), the authors demonstrate that both early child economic well-being as well as caregiver (generally mothers’) education predicts both receptive vocabulary at age 5 years as well as reading comprehension at 15 years. We found this effect is identifiable at an assessment as early as 24 months of age, which is a critical reminder of the enduring impact of these contextual factors on children’s outcomes into young adulthood. The fact that this signal is present at so young an age (typically before most children attend preschool), though an association rather than causal finding, still speaks to the importance of relational factors and that boys may be particularly sensitive to this influence. Furthermore, this is supportive of the global focus on nurturing care to improve early child development and importance of supporting young women to attend and remain in school and the caregiver–child relationship in supporting developmental outcomes [42,43].

Factors that were particularly identified as associated with risk of poor developmental outcomes include those that represent physical health, and these were the factors for which child sex appeared to have had the greatest interaction with developmental outcomes in this cohort. The interaction of male sex with birth weight and developmental outcomes was not a linear one, with boys demonstrating increased vulnerability at lower birth weights but conversely at higher birth weights demonstrating better cognitive and language outcomes. The increased vulnerability of boys to the effects of maternal anaemia and low birth weight may represent a combination of the effects of different timing of brain network development by sex or sex-related adaptation pathways of motor and sensory systems to environmental exposures [8]. Maternal anaemia, one of the pregnancy-related risk factors for which there is robust evidence for long-term developmental impact, came out most strongly as interacting with boys in associating with poor developmental outcomes, though girls were still affected. Though significant only in the unadjusted analysis (and at trend level in the multivariable regression), we mention maternal HIV infection, as it is such an important public health issue. In this cohort, maternal HIV was associated with lower cognitive and language scores in the children. Child HIV infection is known to impact neurodevelopment [44]; however, literature on the impact of maternal HIV exposure without infection is emerging [45,46], and our data indicate that HIV-exposed uninfected children may be affected at 2 years, an area that needs ongoing investigation. We found limited associations with breastfeeding in this analysis; however, it may be due to the relatively low rates of breastfeeding across the cohort, with only 17% of children being exclusively breastfed for 6 months.

Antenatal depression was associated with poorer developmental outcomes across the cohort. The impact of postnatal depression on child growth and development is well established. There is increasing evidence for the effect of prenatal mental disorders including depression on child growth and development, and common mental health disorders, such as depression, appear to increasingly be recognised and highly prevalent in women living in high-risk environments [4,47,48]. Exposure to violence within the home has, likewise, been linked to increased developmental, psychological, and behavioural problems [49–51] as well as impaired child growth both in utero and in early life. Maternal lifetime exposure to IPV increased odds of expressive language delay. Maternal exposure to IPV, depression, or distress may disrupt a mother’s ability to provide care for her child [52], and early childhood adversity has been found to impact child development in other studies [11]. A range of underlying biological mechanisms may be relevant here, including the potential for the infants’ hypothalamic–pituitary axis to have been impacted postnatally through the mother–child interaction [53]. Further work is needed to better explore potential pathways of associations between maternal exposure to violence and child health and developmental outcomes.

Dropout rate remains an important limitation to consider in a study of this type. Of the total live births in the DCHS cohort, approximately 35.8% of children did not attend the 24-month developmental assessment visit (representing 26.8% of the children still in the cohort at 24 months). Although every effort was made to minimise study dropout, the inevitable loss of children from long assessments such as the developmental assessment visit tended to be clustered amongst the slightly younger mothers, who were more likely to be first-time parents and possibly less sensitised to the importance of developmental outcomes. Ex-premature children were also less likely to attend the BSID-III visit. We hypothesise that this may be due to mothers of children being born prematurely having a greater focus on the visits that related to physical health, given the vulnerability of ex-premature infants to intercurrent infections and other physical health problems. It is difficult to interpret the reason for the lower proportion of smoking mothers who chose not to attend the BSID-III visit, although the prevalence of active smoking was high in both groups. Despite these group differences, our remaining sample is large enough and adequately representative of the population in the region for us to be confident that our findings can be meaningfully interpreted. Additionally, bias was reduced by using a population-based cohort study design—choosing a community sample and enrolling consecutive pregnancies when eligible—and performing a complete case–based analysis approach. Finally, the BSID-III uses US population–normative means to create scaled scores, as currently there are no South African norms. Although the BSID-III has been validated for use in South Africa, this limitation means it may not be generalisable to sub-Saharan Africa and may underestimate developmental delay; however, the use of raw scores alongside in this study adds validity to our outcomes.

In conclusion, with the increasing global focus on early child development, population studies directly measuring developmental outcomes are needed to complement the global estimates that use poverty and stunting as proxy measures [41]. This will aid tracking progress towards the Sustainable Development Goals and enable appropriate early child development programmes that are being developed to appropriately target key factors that impact outcomes across the life span. Threats to development, which have been laid out in this manuscript, support the current framework for intervention that targets services for the components of nurturing care [54]. Given the high prevalence of developmental delay in this population, the risk and protective factors identified in this study provide valuable focus for intervention policy design and implementation in this critical area. Public health policy needs to work along the continuum of prepregnancy, pregnancy, and early childhood in the context of families, concentrating on health and nutrition, and encouraging safety, security, and early learning in the context of nurturing care.

## Supporting information

S1 Table Comparison of demographics and baseline characteristics of those children completing at least one domain of the BSID-III at 24 months versus those who were lost to follow-up or missed the 24-month appointment. BSID-III, Bayley-III Scales of Infant and Toddler Development (Third Edition). (DOCX)

S2 Table Bivariate linear regression results demonstrating the association of risk and protective variables with developmental domain raw scores by sex. (DOCX)

S3 Table Bivariate logistic regression results demonstrating the association of risk and protective variables with developmental delay (<−1 SD mean scaled score) by sex. (DOCX)

S4 Table Multivariable logistic regression results demonstrating the association of risk and protective variables with developmental delay by sex. (DOCX)

S5 Table Bivariate logistic regression results demonstrating the association of risk and protective variables with global developmental delay in all four domains for total sample and by sex. (DOCX)

S6 Table Multivariable regression results demonstrating the association of risk and protective variables with developmental delay in all four domains for total sample and exploring interaction with child sex. (DOCX)

S1 Fig Marginal effects of birth weight and sex on receptive language. (TIFF)

## Abbreviations

AIC: Akaike information criterion
aOR: adjusted OR
ASSIST: Alcohol, Smoking and Substance Involvement Screening Test
BSID-III: Bayley-III Scales of Infant and Toddler Development (Third Edition)
CI: confidence interval
DCHS: Drakenstein Child Health Study
EPDS: Edinburgh Postnatal Depression Scale
IPV: intimate partner violence
LMIC: low- and middle-income country
SASH: South African Stress and Health
SRQ-20: Self Reporting Questionnaire-20
