# Supplementary material for: Risk and protective factors for child development: An observational South African birth cohort
Source: PLoS Med. 2019 Sep 27;16(9):e1002920. doi: 10.1371/journal.pmed.1002920 (PMC6764658; doi:10.1371/journal.pmed.1002920)
Supplement: S1 Table — (DOCX) [file pmed.1002920.s001.docx]

**S1 Table:** Comparison of demographics and baseline characteristics of those children completing at least one domain of the BSID-III at 24 months versus those who were lost to follow up or missed the 24-month appointment.

|  | BSID-III completed | No BSID-III | Total | *P* |
| --- | --- | --- | --- | --- |
| Total | 734 (64.22) | 409 (35.78) | 1143 (100) |  |
| Child sex: boys | 380 (51.77) | 210 (51.57) | 590 (51.66) | 0.92 |
| Maternal education: secondary and above | 674 (91.83) | 383 (93.64) | 1057 (92.05) | 0.26 |
| Socioeconomic |  |  |  |  |
| Household Income: > R1000 per month | 447 (60.90) | 265 (64.79) | 712 (62.29) | 0.19 |
| Tap Running Water | 506 (69.22) | 283 (69.53) | 789 (69.33) | 0.91 |
| Flush Toilet | 466 (63.66) | 257 (63.14) | 723 (63.48) | 0.80 |
| Electricity | 695 (94.95) | 376 (92.38) | 1071 (94.03) | 0.08 |
| Maternal Age at Enrolment (mean, SD) | 26.97 + (5.83) | 25.94 + (5.35) | 26.60 + (5.68) | <0.01* |
| Married or Cohabitating | 294 (40.11) | 168 (41.08) | 462 (40.46) | 0.75 |
| Employed | 183 (24.93) | 124 (30.32) | 307 (26.86) | 0.05* |
| Primigravida | 242 (32.97) | 157 (38.39) | 399 (34.91) | 0.07 |
| Physical |  |  |  |  |
| Preterm | 104 (14.21) | 84 (20.69) | 188 (16.52) | <0.01* |
| Birthweight (kg) (mean, SD) | 3.03 + (0.58) | 3.00 + (0.65) | 3.02 + (0.61) | 0.95 |
| Exclusive Breastfeeding for 6 months | 125 (17.05) | 43 (12.91) | 168 (15.76) | 0.09 |
| Maternal HIV infection | 169 (23.02) | 78 (19.07) | 247 (21.61) | 0.12 |
| Maternal anaemia in pregnancy | 116 (17.06) | 65 (16.54) | 181 (16.87) | 0.83 |
| Maternal alcohol use in pregnancy | 95 (14.48) | 36 (10.59) | 131 (13.15) | 0.09 |
| Maternal active smoking in pregnancy | 241 (34.73) | 100 (27.25) | 341 (32.14) | 0.01* |
| Psychosocial |  |  |  |  |
| Antenatal depression | 156 (23.71) | 82 (24.05) | 238 (23.82) | 0.91 |
| Antenatal psychological distress | 138 (20.97) | 65 (19.01) | 203 (20.30) | 0.46 |
| Lifetime intimate partner violence | 310 (47.26) | 144 (42.73) | 454 (45.72) | 0.18 |
| Maternal childhood trauma | 228 (34.65) | 115 (33.72) | 343 (34.33) | 0.77 |

***Footnotes:***

* p<0.05; Mann-Whitney U tests used for continuous variables (means and SD presented); Chi-squared for categorical variables (n and % proportions presented).
