# Supplementary material for: Risk and protective factors for child development: An observational South African birth cohort
Source: PLoS Med. 2019 Sep 27;16(9):e1002920. doi: 10.1371/journal.pmed.1002920 (PMC6764658; doi:10.1371/journal.pmed.1002920)
Supplement: S2 Table — (DOCX) [file pmed.1002920.s002.docx]

**S2 Table: Bivariate linear regression results demonstrating the association of risk and protective variables with developmental domain raw scores by sex.**

|  | **Cognitive** | | | **Receptive** | | | **Expressive** | | | **Fine Motor** | | |
| --- | --- | --- | --- | --- | --- | --- | --- | --- | --- | --- | --- | --- |
| **VARIABLES** | **Total** | **Females** | **Males** | **Total** | **Females** | **Males** | **Total** | **Females** | **Males** | **Total** | **Females** | **Males** |
| **A priori variables** |  |  |  |  |  |  |  |  |  |  |  |  |
| Education: >=Secondary | 2.03  (0.76 ; 3.30) | 1.06  (-0.76 ; 2.89) | 2.86  (1.10 ; 4.62) | 1.16  (0.17 ; 2.16) | 0.76  (-0.72 ; 2.24) | 1.48  (0.16 ; 2.80) | 2.08  (0.72 ; 3.44) | 1.62  (-0.46 ; 3.69) | 2.35  (0.60 ; 4.10) | 0.86  (0.02 ; 1.69) | 0.74  (-0.44 ; 1.91) | 0.95  (-0.23 ; 2.13) |
| Child Age | 0.73  (0.06 ; 1.40) | 1.01  (0.02 ; 2.01) | 0.52  (-0.39 ; 1.42) | 0.53  (0.01 ; 1.05) | 0.57  (-0.23 ; 1.37) | 0.48  (-0.19 ; 1.16) | 0.99  (0.27 ; 1.71) | 1.00  (-0.11 ; 2.11) | 0.96  (0.05 ; 1.88) | 0.41  (-0.03 ; 0.85) | 0.68  (0.03 ; 1.33) | 0.22  (-0.38 ; 0.82) |
| Child Sex: Boys | -0.75  (-1.45 ; -0.05) |  |  | -1.18  (-1.72 ; -0.65) |  |  | -1.76  (-2.50 ; -1.01) |  |  | -0.67  (-1.13 ; -0.21) |  |  |
| **Socioeconomic** |  |  |  |  |  |  |  |  |  |  |  |  |
| Household Income: >R1000 per month | 0.54  (-0.18 ; 1.26) | 1.07  (0.04 ; 2.11) | -0.04  (-1.04 ; 0.96) | 0.63  (0.07 ; 1.18) | 0.43  (-0.41 ; 1.26) | 0.58  (-0.15 ; 1.32) | 0.96  (0.19 ; 1.73) | 1.16  (-0.01 ; 2.33) | 0.48  (-0.53 ; 1.48) | 0.33  (-0.14 ; 0.80) | 0.63  (-0.04 ; 1.30) | -0.05  (-0.71 ; 0.62) |
| Tap Running Water | 0.40  (-0.36 ; 1.16) | 0.15  (-0.96 ; 1.25) | 0.53  (-0.53 ; 1.59) | 0.50  (-0.09 ; 1.09) | 0.10  (-0.79 ; 0.99) | 0.69  (-0.09 ; 1.46) | 0.27  (-0.55 ; 1.09) | -0.10  (-1.35 ; 1.15) | 0.36  (-0.71 ; 1.42) | 0.25  (-0.25 ; 0.75) | 0.51  (-0.20 ; 1.23) | -0.05  (-0.75 ; 0.65) |
| Flush toilet | 0.52  (-0.21 ; 1.25) | 0.49  (-0.56 ; 1.54) | 0.45  (-0.56 ; 1.47) | 0.58  (0.01 ; 1.14) | 0.36  (-0.49 ; 1.20) | 0.62  (-0.13 ; 1.36) | 0.98  (0.20 ; 1.76) | 1.07  (-0.11 ; 2.25) | 0.69  (-0.34 ; 1.71) | 0.31  (-0.17 ; 0.78) | 0.43  (-0.25 ; 1.11) | 0.12  (-0.55 ; 0.79) |
| Electricity | 1.37  (-0.23 ; 2.97) | 2.22  (-0.02 ; 4.45) | 0.58  (-1.70 ; 2.85) | 0.99  (-0.26 ; 2.24) | 1.30  (-0.53 ; 3.14) | 0.69  (-0.98 ; 2.36) | 0.84  (-0.92 ; 2.59) | 1.64  (-0.97 ; 4.25) | 0.09  (-2.22 ; 2.40) | 0.30  (-0.77 ; 1.36) | 1.05  (-0.43 ; 2.53) | -0.39  (-1.90 ; 1.11) |
| Maternal Age at Enrolment | -0.07  (-0.13 ; -0.01) | -0.09  (-0.18 ; -0.01) | -0.05  (-0.14 ; 0.03) | -0.03  (-0.08 ; 0.02) | -0.06  (-0.13 ; 0.00) | -0.00  (-0.07 ; 0.06) | -0.04  (-0.11 ; 0.02) | -0.09  (-0.19 ; 0.00) | -0.01  (-0.09 ; 0.08) | -0.02  (-0.06 ; 0.02) | -0.02  (-0.08 ; 0.03) | -0.02  (-0.07 ; 0.04) |
| Married/cohabitating | -0.24  (-0.96 ; 0.47) | -0.10  (-1.10 ; 0.90) | -0.48  (-1.50 ; 0.55) | -0.28  (-0.84 ; 0.27) | -0.74  (-1.53 ; 0.06) | 0.02  (-0.74 ; 0.77) | -0.21  (-0.98 ; 0.56) | -0.59  (-1.71 ; 0.54) | -0.08  (-1.12 ; 0.96) | -0.11  (-0.58 ; 0.36) | -0.18  (-0.83 ; 0.46) | -0.13  (-0.80 ; 0.55) |
| Employed | 0.36  (-0.45 ; 1.17) | 0.49  (-0.65 ; 1.63) | 0.22  (-0.93 ; 1.37) | 0.53  (-0.10 ; 1.16) | 0.51  (-0.40 ; 1.42) | 0.52  (-0.34 ; 1.37) | 0.13  (-0.74 ; 1.00) | 0.12  (-1.16 ; 1.39) | 0.11  (-1.05 ; 1.27) | 0.35  (-0.18 ; 0.88) | 0.59  (-0.14 ; 1.32) | 0.11  (-0.65 ; 0.87) |
| Primigravid | 0.67  (-0.07 ; 1.42) | 1.16  (0.09 ; 2.22) | 0.30  (-0.74 ; 1.35) | 0.77  (0.20 ; 1.35) | 1.36  (0.52 ; 2.20) | 0.31  (-0.46 ; 1.08) | 1.19  (0.39 ; 1.98) | 1.93  (0.76 ; 3.10) | 0.61  (-0.44 ; 1.66) | 0.16  (-0.32 ; 0.65) | 0.43  (-0.25 ; 1.12) | -0.03  (-0.72 ; 0.66) |
| **Physical** |  |  |  |  |  |  |  |  |  |  |  |  |
| Preterm | -1.79  (-2.78 ; -0.79) | -2.60  (-4.01 ; -1.20) | -1.04  (-2.44 ; 0.36) | -0.68  (-1.46 ; 0.09) | -0.78  (-1.92 ; 0.37) | -0.56  (-1.59 ; 0.48) | -1.08  (-2.17 ; 0.02) | -1.38  (-3.02 ; 0.25) | -0.73  (-2.16 ; 0.70) | -0.60  (-1.26 ; 0.06) | -1.16  (-2.09 ; -0.24) | -0.08  (-1.02 ; 0.85) |
| Birthweight | 1.55  (0.95 ; 2.14) | 1.07  (0.21 ; 1.92) | 1.97  (1.15 ; 2.80) | 0.94  (0.47 ; 1.41) | 0.29  (-0.41 ; 0.99) | 1.50  (0.89 ; 2.10) | 1.04  (0.38 ; 1.69) | 0.58  (-0.42 ; 1.59) | 1.37  (0.54 ; 2.20) | 0.76  (0.36 ; 1.15) | 0.67  (0.11 ; 1.22) | 0.83  (0.27 ; 1.38) |
| Exclusive Breastfeeding for 6 months | -0.24  (-1.17 ; 0.70) | 0.38  (-0.94 ; 1.70) | -0.82  (-2.14 ; 0.50) | 0.41  (-0.32 ; 1.13) | 0.98  (-0.06 ; 2.03) | -0.17  (-1.16 ; 0.81) | 0.21  (-0.79 ; 1.22) | 1.25  (-0.23 ; 2.73) | -0.73  (-2.06 ; 0.60) | -0.14  (-0.75 ; 0.47) | 0.23  (-0.62 ; 1.08) | -0.50  (-1.37 ; 0.38) |
| Maternal HIV infection | -0.86  (-1.69 ; -0.03) | -1.08  (-2.29 ; 0.14) | -0.60  (-1.74 ; 0.55) | -1.27  (-1.91 ; -0.64) | -1.17  (-2.14 ; -0.19) | -1.22  (-2.05 ; -0.39) | -1.56  (-2.45 ; -0.67) | -1.89  (-3.27 ; -0.52) | -1.07  (-2.22 ; 0.07) | -0.13  (-0.68 ; 0.42) | -0.37  (-1.16 ; 0.42) | 0.14  (-0.62 ; 0.89) |
| Maternal anaemia in pregnancy | -0.62  (-1.59 ; 0.35) | -1.11  (-2.42 ; 0.21) | -0.16  (-1.60 ; 1.27) | -0.96  (-1.71 ; -0.21) | -1.62  (-2.67 ; -0.57) | -0.36  (-1.41 ; 0.69) | -1.06  (-2.10 ; -0.02) | -2.03  (-3.50 ; -0.55) | -0.22  (-1.65 ; 1.21) | 0.13  (-0.50 ; 0.77) | -0.11  (-0.96 ; 0.75) | 0.32  (-0.61 ; 1.25) |
| Maternal alcohol use in pregnancy | -0.28  (-1.34 ; 0.78) | -0.45  (-2.03 ; 1.12) | -0.09  (-1.52 ; 1.35) | -0.14  (-0.97 ; 0.69) | -0.41  (-1.69 ; 0.86) | 0.19  (-0.87 ; 1.26) | -0.35  (-1.50 ; 0.80) | -0.01  (-1.76 ; 1.75) | -0.47  (-1.97 ; 1.03) | -0.21  (-0.90 ; 0.48) | -0.92  (-1.91 ; 0.08) | 0.42  (-0.54 ; 1.37) |
| Maternal active smoking in pregnancy | -0.54  (-1.28 ; 0.19) | -0.85  (-1.88 ; 0.19) | -0.23  (-1.27 ; 0.80) | -0.14  (-0.73 ; 0.44) | -0.43  (-1.28 ; 0.42) | 0.17  (-0.61 ; 0.96) | -0.51  (-1.33 ; 0.30) | -0.33  (-1.53 ; 0.87) | -0.58  (-1.66 ; 0.50) | -0.41  (-0.89 ; 0.07) | -0.90  (-1.57 ; -0.23) | 0.05  (-0.63 ; 0.74) |
| **Psychosocial** |  |  |  |  |  |  |  |  |  |  |  |  |
| Antenatal depression | -0.86  (-1.73 ; 0.01) | -0.17  (-1.41 ; 1.07) | -1.58  (-2.80 ; -0.36) | -0.54  (-1.22 ; 0.14) | -0.29  (-1.28 ; 0.70) | -0.87  (-1.79 ; 0.05) | -1.06  (-1.99 ; -0.13) | -0.26  (-1.61 ; 1.09) | -1.96  (-3.22 ; -0.70) | -0.13  (-0.70 ; 0.45) | 0.23  (-0.56 ; 1.02) | -0.52  (-1.34 ; 0.30) |
| Antenatal psychological distress | -0.16  (-1.08 ; 0.75) | 0.51  (-0.76 ; 1.78) | -0.97  (-2.29 ; 0.35) | -0.16  (-0.87 ; 0.56) | -0.15  (-1.17 ; 0.87) | -0.32  (-1.30 ; 0.67) | -0.33  (-1.31 ; 0.65) | 0.08  (-1.31 ; 1.47) | -1.01  (-2.37 ; 0.36) | -0.16  (-0.76 ; 0.44) | -0.29  (-1.09 ; 0.52) | -0.13  (-1.01 ; 0.75) |
| Maternal Childhood Trauma | -0.09  (-0.88 ; 0.69) | -0.32  (-1.45 ; 0.80) | 0.11  (-0.98 ; 1.20) | -0.24  (-0.85 ; 0.37) | -0.28  (-1.18 ; 0.62) | -0.19  (-1.00 ; 0.62) | -0.41  (-1.25 ; 0.43) | -0.07  (-1.30 ; 1.15) | -0.75  (-1.88 ; 0.37) | 0.08  (-0.44 ; 0.59) | -0.29  (-1.00 ; 0.43) | 0.40  (-0.32 ; 1.12) |
| Lifetime intimate partner violence | -0.05  (-0.79 ; 0.70) | -0.25  (-1.33 ; 0.83) | 0.18  (-0.86 ; 1.22) | -0.20  (-0.78 ; 0.38) | -0.32  (-1.18 ; 0.54) | -0.01  (-0.78 ; 0.76) | 0.07  (-0.73 ; 0.87) | -0.33  (-1.51 ; 0.84) | 0.53  (-0.54 ; 1.60) | 0.19  (-0.30 ; 0.68) | 0.14  (-0.55 ; 0.83) | 0.28  (-0.41 ; 0.97) |

***Footnotes***

Green signifies a positive association with p<0.05; red signifies a negative association with p<0.05

Coefficients and 95% confidence intervals presented for variables in each model.
