# Supplementary material for: Risk and protective factors for child development: An observational South African birth cohort
Source: PLoS Med. 2019 Sep 27;16(9):e1002920. doi: 10.1371/journal.pmed.1002920 (PMC6764658; doi:10.1371/journal.pmed.1002920)
Supplement: S3 Table — (DOCX) [file pmed.1002920.s003.docx]

**S3 Table: Bivariate logistic regression results demonstrating the association of risk and protective variables with developmental delay (<-1 SD mean scaled score) by sex.**

|  | **Cognitive** | | | **Receptive** | | | **Expressive** | | | **Fine Motor** | | |
| --- | --- | --- | --- | --- | --- | --- | --- | --- | --- | --- | --- | --- |
| **VARIABLES** | **Total** | **Females** | **Males** | **Total** | **Females** | **Males** | **Total** | **Females** | **Males** | **Total** | **Females** | **Males** |
| **A priori variables** |  |  |  |  |  |  |  |  |  |  |  |  |
| Education: >=Secondary | 0.56  (0.33 ; 0.97) | 0.63  (0.29 ; 1.38) | 0.51  (0.23 ; 1.10) | 0.45  (0.25 ; 0.82) | 0.55  (0.25 ; 1.22) | 0.33  (0.12 ; 0.88) | 0.74  (0.43 ; 1.29) | 1.03  (0.46 ; 2.30) | 0.55  (0.24 ; 1.26) | 0.62  (0.35 ; 1.10) | 0.66  (0.27 ; 1.62) | 0.60  (0.28 ; 1.27) |
| Child Age | 1.20  (0.91 ; 1.59) | 1.01  (0.66 ; 1.54) | 1.39  (0.94 ; 2.05) | 1.07  (0.80 ; 1.42) | 0.99  (0.64 ; 1.51) | 1.17  (0.78 ; 1.75) | 0.94  (0.70 ; 1.25) | 0.94  (0.61 ; 1.44) | 0.94  (0.64 ; 1.39) | 0.97  (0.69 ; 1.35) | 1.34  (0.78 ; 2.30) | 0.79  (0.51 ; 1.22) |
| Child Sex: Boys | 1.35  (1.01 ; 1.81) |  |  | 2.22  (1.64 ; 2.99) |  |  | 1.95  (1.44 ; 2.64) |  |  | 1.65  (1.16 ; 2.35) |  |  |
| **Socioeconomic** |  |  |  |  |  |  |  |  |  |  |  |  |
| Household Income: >R1000 per month | 0.67  (0.49 ; 0.90) | 0.61  (0.39 ; 0.96) | 0.75  (0.50 ; 1.12) | 0.80  (0.59 ; 1.08) | 0.91  (0.59 ; 1.42) | 0.81  (0.53 ; 1.24) | 0.75  (0.55 ; 1.02) | 0.85  (0.54 ; 1.34) | 0.75  (0.48 ; 1.15) | 0.79  (0.56 ; 1.12) | 0.49  (0.28 ; 0.85) | 1.19  (0.75 ; 1.88) |
| Tap Running Water | 0.64  (0.47 ; 0.89) | 0.82  (0.51 ; 1.31) | 0.54  (0.35 ; 0.83) | 0.67  (0.48 ; 0.92) | 0.82  (0.51 ; 1.31) | 0.59  (0.37 ; 0.94) | 0.96  (0.70 ; 1.33) | 1.20  (0.74 ; 1.94) | 0.87  (0.55 ; 1.37) | 0.70  (0.49 ; 1.01) | 0.54  (0.31 ; 0.96) | 0.87  (0.54 ; 1.40) |
| Flush toilet | 0.69  (0.51 ; 0.93) | 0.83  (0.53 ; 1.29) | 0.60  (0.40 ; 0.92) | 0.63  (0.46 ; 0.86) | 0.72  (0.46 ; 1.13) | 0.59  (0.38 ; 0.93) | 0.85  (0.62 ; 1.16) | 0.94  (0.60 ; 1.48) | 0.83  (0.54 ; 1.29) | 0.68  (0.48 ; 0.97) | 0.50  (0.29 ; 0.86) | 0.89  (0.56 ; 1.41) |
| Electricity | 0.60  (0.30 ; 1.19) | 0.68  (0.26 ; 1.77) | 0.52  (0.20 ; 1.41) | 0.53  (0.26 ; 1.10) | 0.33  (0.11 ; 0.95) | 0.85  (0.31 ; 2.28) | 0.50  (0.24 ; 1.06) | 0.38  (0.13 ; 1.12) | 0.65  (0.23 ; 1.86) | 1.06  (0.47 ; 2.37) | 0.53  (0.18 ; 1.56) | 2.08  (0.59 ; 7.29) |
| Maternal Age at Enrolment | 1.02  (0.99 ; 1.04) | 1.02  (0.98 ; 1.06) | 1.01  (0.98 ; 1.05) | 1.01  (0.98 ; 1.03) | 1.01  (0.97 ; 1.04) | 1.01  (0.97 ; 1.04) | 1.01  (0.98 ; 1.03) | 1.03  (0.99 ; 1.07) | 0.99  (0.95 ; 1.02) | 1.00  (0.98 ; 1.03) | 1.01  (0.96 ; 1.05) | 1.01  (0.97 ; 1.04) |
| Married/cohabitating | 1.03  (0.77 ; 1.39) | 1.07  (0.70 ; 1.63) | 1.03  (0.68 ; 1.57) | 1.10  (0.81 ; 1.48) | 1.33  (0.87 ; 2.03) | 1.00  (0.65 ; 1.55) | 1.12  (0.83 ; 1.52) | 1.33  (0.86 ; 2.05) | 1.04  (0.67 ; 1.62) | 1.04  (0.73 ; 1.48) | 1.09  (0.63 ; 1.87) | 1.07  (0.67 ; 1.70) |
| Employed | 0.88  (0.63 ; 1.23) | 0.86  (0.53 ; 1.40) | 0.90  (0.56 ; 1.44) | 0.89  (0.63 ; 1.24) | 0.91  (0.56 ; 1.48) | 0.87  (0.54 ; 1.43) | 1.05  (0.74 ; 1.48) | 1.18  (0.72 ; 1.93) | 0.94  (0.58 ; 1.54) | 1.03  (0.69 ; 1.52) | 0.86  (0.45 ; 1.61) | 1.17  (0.70 ; 1.96) |
| Primigravid | 0.79  (0.58 ; 1.07) | 0.61  (0.38 ; 0.96) | 0.96  (0.63 ; 1.47) | 0.76  (0.56 ; 1.04) | 0.67  (0.42 ; 1.05) | 0.81  (0.52 ; 1.26) | 0.68  (0.49 ; 0.93) | 0.47  (0.29 ; 0.75) | 0.90  (0.57 ; 1.40) | 0.81  (0.56 ; 1.18) | 0.60  (0.32 ; 1.12) | 0.95  (0.59 ; 1.53) |
| **Physical** |  |  |  |  |  |  |  |  |  |  |  |  |
| Preterm | 1.69  (1.10 ; 2.58) | 1.82  (0.98 ; 3.36) | 1.57  (0.87 ; 2.84) | 1.51  (0.98 ; 2.32) | 1.50  (0.82 ; 2.77) | 1.51  (0.80 ; 2.85) | 1.48  (0.95 ; 2.30) | 1.43  (0.76 ; 2.71) | 1.50  (0.79 ; 2.85) | 1.92  (1.22 ; 3.01) | 2.66  (1.35 ; 5.24) | 1.48  (0.81 ; 2.73) |
| Birthweight | 0.57  (0.43 ; 0.74) | 0.80  (0.55 ; 1.15) | 0.40  (0.27 ; 0.59) | 0.61  (0.46 ; 0.79) | 0.78  (0.53 ; 1.13) | 0.45  (0.30 ; 0.68) | 0.65  (0.49 ; 0.85) | 0.85  (0.57 ; 1.25) | 0.49  (0.33 ; 0.73) | 0.55  (0.41 ; 0.74) | 0.49  (0.31 ; 0.77) | 0.61  (0.41 ; 0.89) |
| Exclusive Breastfeeding for 6 months | 1.06  (0.72 ; 1.56) | 0.93  (0.53 ; 1.63) | 1.20  (0.70 ; 2.06) | 0.82  (0.56 ; 1.21) | 0.59  (0.33 ; 1.04) | 1.16  (0.65 ; 2.07) | 0.97  (0.65 ; 1.43) | 0.80  (0.45 ; 1.42) | 1.16  (0.65 ; 2.06) | 1.07  (0.68 ; 1.68) | 0.98  (0.48 ; 2.00) | 1.15  (0.64 ; 2.07) |
| Maternal HIV infection | 1.29  (0.91 ; 1.82) | 1.07  (0.64 ; 1.79) | 1.46  (0.91 ; 2.35) | 1.51  (1.05 ; 2.15) | 1.39  (0.83 ; 2.35) | 1.50  (0.90 ; 2.49) | 1.14  (0.80 ; 1.63) | 0.97  (0.57 ; 1.66) | 1.19  (0.73 ; 1.96) | 1.15  (0.77 ; 1.72) | 1.07  (0.56 ; 2.07) | 1.15  (0.69 ; 1.92) |
| Maternal anaemia in pregnancy | 1.43  (0.95 ; 2.14) | 1.73  (0.99 ; 3.04) | 1.19  (0.66 ; 2.15) | 1.53  (1.01 ; 2.33) | 2.30  (1.30 ; 4.08) | 1.05  (0.57 ; 1.95) | 1.55  (1.01 ; 2.37) | 1.85  (1.04 ; 3.29) | 1.39  (0.73 ; 2.67) | 1.04  (0.65 ; 1.65) | 1.19  (0.60 ; 2.35) | 0.97  (0.51 ; 1.85) |
| Maternal alcohol use in pregnancy | 1.12  (0.72 ; 1.73) | 1.19  (0.62 ; 2.27) | 1.04  (0.58 ; 1.88) | 0.82  (0.53 ; 1.27) | 0.86  (0.44 ; 1.68) | 0.71  (0.39 ; 1.30) | 1.11  (0.70 ; 1.76) | 0.96  (0.48 ; 1.89) | 1.20  (0.63 ; 2.29) | 1.63  (1.01 ; 2.65) | 4.13  (2.04 ; 8.37) | 0.76  (0.38 ; 1.52) |
| Maternal active smoking in pregnancy | 1.31  (0.96 ; 1.80) | 1.41  (0.89 ; 2.24) | 1.21  (0.79 ; 1.87) | 1.00  (0.73 ; 1.37) | 1.12  (0.71 ; 1.77) | 0.85  (0.54 ; 1.33) | 1.11  (0.81 ; 1.54) | 1.08  (0.67 ; 1.72) | 1.12  (0.71 ; 1.77) | 0.97  (0.66 ; 1.41) | 1.66  (0.93 ; 2.96) | 0.64  (0.39 ; 1.07) |
| **Psychosocial** |  |  |  |  |  |  |  |  |  |  |  |  |
| Antenatal depression | 1.43  (1.00 ; 2.06) | 1.22  (0.73 ; 2.03) | 1.73  (1.03 ; 2.92) | 1.16  (0.80 ; 1.68) | 1.13  (0.67 ; 1.89) | 1.29  (0.74 ; 2.24) | 1.61  (1.10 ; 2.36) | 1.51  (0.89 ; 2.55) | 1.88  (1.05 ; 3.35) | 0.99  (0.64 ; 1.52) | 1.43  (0.75 ; 2.71) | 0.76  (0.42 ; 1.37) |
| Antenatal psychological distress | 1.06  (0.73 ; 1.55) | 0.70  (0.41 ; 1.20) | 1.74  (0.99 ; 3.04) | 1.14  (0.77 ; 1.67) | 1.04  (0.61 ; 1.76) | 1.45  (0.79 ; 2.63) | 1.21  (0.81 ; 1.79) | 1.05  (0.61 ; 1.80) | 1.60  (0.87 ; 2.95) | 0.84  (0.53 ; 1.33) | 1.28  (0.66 ; 2.48) | 0.62  (0.32 ; 1.20) |
| Maternal Childhood Trauma | 1.03  (0.74 ; 1.42) | 1.22  (0.77 ; 1.94) | 0.88  (0.56 ; 1.37) | 1.21  (0.87 ; 1.68) | 1.33  (0.83 ; 2.13) | 1.11  (0.69 ; 1.79) | 1.18  (0.84 ; 1.64) | 1.14  (0.71 ; 1.83) | 1.24  (0.77 ; 2.00) | 1.02  (0.69 ; 1.49) | 1.71  (0.94 ; 3.08) | 0.70  (0.42 ; 1.18) |
| Lifetime intimate partner violence | 0.98  (0.72 ; 1.34) | 0.93  (0.59 ; 1.45) | 1.02  (0.67 ; 1.56) | 1.01  (0.74 ; 1.38) | 1.28  (0.81 ; 2.00) | 0.75  (0.48 ; 1.18) | 1.38  (1.01 ; 1.90) | 1.73  (1.09 ; 2.73) | 1.09  (0.69 ; 1.71) | 0.76  (0.53 ; 1.10) | 0.98  (0.55 ; 1.76) | 0.62  (0.38 ; 1.01) |

***Footnotes:***

Green signifies a decreased risk of delay with p<0.05; red signifies an increased risk of delay with p<0.05

Odd’s ratios and 95% confidence intervals presented for variables in each model.
