# Supplementary material for: Risk and protective factors for child development: An observational South African birth cohort
Source: PLoS Med. 2019 Sep 27;16(9):e1002920. doi: 10.1371/journal.pmed.1002920 (PMC6764658; doi:10.1371/journal.pmed.1002920)
Supplement: S5 Table — (DOCX) [file pmed.1002920.s005.docx]

**S5 Table: Bivariate logistic regression results demonstrating the association of risk and protective variables with global developmental delay in all 4 domains for total sample and by sex.**

|  | **Global developmental delay** | | |
| --- | --- | --- | --- |
|  | **Total** | **Girls** | **Boys** |
| **A priori variables** |  |  |  |
| Education: >=Secondary | 0.47  (0.23; 0.95) | 0.42  (0.15; 1.18) | 0.52  (0.20; 1.33) |
| Child Age | 1.03  (0.66; 1.63 | 1.24  (0.60; 2.54) | 0.92  (0.51; 1.68) |
| Child Sex: Boys | 1.28  (0.79; 2.07) |  |  |
| **Socioeconomic** |  |  |  |
| Household Income: > R1000 per month | 0.63  (0.39; 1.02) | 0.32  (0.15; 0.68) | 1.07  (0.56; 2.04) |
| Tap Running Water | 0.72  (0.44; 1.18) | 0.71  (0.33; 1.54) | 0.74  (0.38; 1.42) |
| Flush Toilet | 0.70  (0.43; 1.14) | 0.53  (0.25; 1.10) | 0.89  (0.47; 1.69) |
| Electricity | 0.94  (0.32; 2.73) | 0.48  (0.13; 1.74) | 2.38  (0.31; 18.27) |
| Maternal Age at Enrolment | 0.98  (0.94; 1.02) | 0.96  (0.90; 1.03) | 0.99  (0.94; 1.05) |
| Married or cohabitating | 1.13  (0.70; 1.83) | 0.78  (0.37; 1.65) | 1.53  (0.81; 2.90) |
| Maternal employment | 0.54  (0.29; 1.03) | 0.52  (0.19; 1.40) | 0.56  (0.24; 1.30) |
| Primigravid | 1.02  (0.61; 1.69) | 0.84  (0.38; 1.88) | 1.14  (0.59; 2.21) |
| **Physical** |  |  |  |
| Birthweight | 0.56  (0.38; 0.81) | 0.53  (0.30; 0.93) | 0.58  (0.34; 0.97) |
| Preterm | 2.28  (1.29; 4.02) | 3.28  (1.44; 7.43) | 1.67  (0.75; 3.71) |
| Exclusive Breastfeeding for 6 months | 1.26  (0.69; 2.29) | 0.89  (0.33; 2.42) | 1.59  (0.74; 3.41) |
| Maternal HIV infection | 1.35  (0.79; 2.31) | 1.08  (0.45; 2.61) | 1.53  (0.77; 3.03) |
| Maternal anaemia in pregnancy | 1.09  (0.58; 2.06) | 1.34  (0.55; 3.27) | 0.92  (0.37; 2.30) |
| Maternal alcohol use in pregnancy | 1.65  (0.88; 3.12) | 3.13  (1.27; 7.71) | 0.95  (0.38; 2.40) |
| Maternal active smoking in pregnancy | 1.03  (0.61; 1.73 | 1.79  (0.80; 4.02) | 0.68  (0.34; 1.38) |
| **Psychosocial** |  |  |  |
| Antenatal Depression | 1.10  (0.62; 1.97) | 1.06  (0.43; 2.62) | 1.15  (0.54; 2.48) |
| Antenatal psychological distress | 1.10  (0.60; 2.01) | 1.44  (0.60; 3.44) | 0.90  (0.38; 2.13) |
| Lifetime intimate partner violence | 0.98  (0.59; 1.64) | 0.80  (0.36; 1.79) | 1.12  (0.57; 2.18) |
| Maternal childhood trauma | 1.40  (0.84; 2.34) | 1.85  (0.84; 4.09) | 1.15  (0.58; 2.27) |

***Footnotes:***

Only variables included in the stepwise regression models are shown Green signifies a decreased risk of delay with p<0.05; red signifies an increased risk of delay with p<0.05; Adjusted odd’s ratios and 95% confidence intervals presented for variables in each model.
