# Supplementary material for: Risk and protective factors for child development: An observational South African birth cohort
Source: PLoS Med. 2019 Sep 27;16(9):e1002920. doi: 10.1371/journal.pmed.1002920 (PMC6764658; doi:10.1371/journal.pmed.1002920)
Supplement: S6 Table — (DOCX) [file pmed.1002920.s006.docx]

**S6 Table: Multivariable logistic regression results demonstrating the association of risk and protective variables with global developmental delay in all domains and the interaction with child sex.**

|  | **Final model** | **Interaction with child sex model** |
| --- | --- | --- |
| **A priori variables** |  |  |
| Education: >=Secondary | 0.40  (0.17 ; 0.94) | 0.40  (0.17 ; 0.95) |
| Child Age | 1.07  (0.61 ; 1.86) | 1.08  (0.62 ; 1.88) |
| Child Sex: Boys | 1.82  (0.98 ; 3.38) | 2.08  (1.07 ; 4.05) |
| **Socioeconomic** |  |  |
| Household Income: > R1000 per month |  |  |
| Tap Running Water |  |  |
| Flush Toilet |  |  |
| Electricity |  |  |
| Maternal Age | 0.95  (0.90 ; 1.00) | 0.89  (0.81 ; 0.99)*  1.10  (0.97 ; 1.23)** |
| Married or Cohabitating |  |  |
| Employed |  |  |
| Primigravid |  |  |
| **Physical** |  |  |
| Birthweight |  |  |
| Preterm |  |  |
| Exclusive Breastfeeding for 6 months |  |  |
| Maternal HIV infection |  |  |
| Maternal anaemia in pregnancy |  |  |
| Maternal alcohol use in pregnancy |  |  |
| Maternal active smoking in pregnancy |  |  |
| **Psychosocial** |  |  |
| Antenatal Depression |  |  |
| Antenatal psychological distress |  |  |
| Lifetime intimate partner violence |  |  |
| Maternal childhood trauma |  |  |

***Footnotes:***

The ‘final model’ describes the multivariable model assessing associations of risk and protective factors and developmental outcomes.

The ‘interaction with child sex model’ explored the interaction with child sex for each variable included in the final model, and shows the model inclusive of those interactions that reduced the AIC.

* beta coefficient from the interaction model of the variable main effect

** beta coefficient from the interaction model of the variable with male sex

The complete case dataset was used here (n=539).

Green signifies a positive association with p<0.05; red signifies a negative association with p<0.05

Adjusted odd’s ratios and 95% confidence intervals presented for variables in each model
