# Supplementary figures and images for: Risk and protective factors for child development: An observational South African birth cohort
Source: PLoS Med. 2019 Sep 27;16(9):e1002920. doi: 10.1371/journal.pmed.1002920 (PMC6764658; doi:10.1371/journal.pmed.1002920)

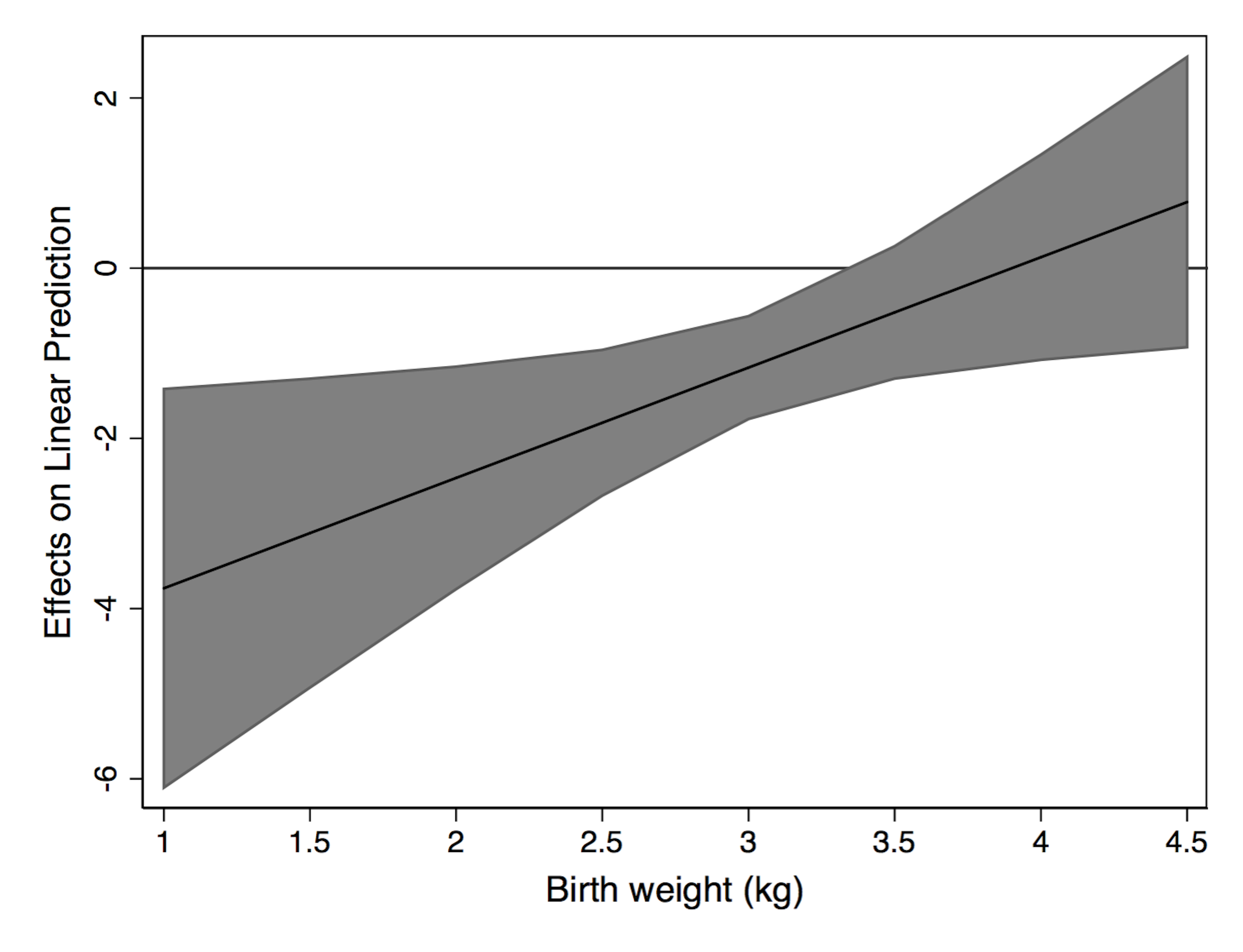

Supplement: S1 Fig — (TIFF) [file pmed.1002920.s007.tiff]
